# Design and synthesis of fused polycycles via Diels–Alder reaction and ring-rearrangement metathesis as key steps

**DOI:** 10.3762/bjoc.11.140

**Published:** 2015-07-27

**Authors:** Sambasivarao Kotha, Ongolu Ravikumar

**Affiliations:** 1 Department of Chemistry, Indian Institute of Technology-Bombay, Powai, Mumbai-400 076, India, Fax: 022-25767152

**Keywords:** Diels–Alder reaction, Grignard addition, ring-rearrangement metathesis, polycycles

## Abstract

Atom efficient processes such as the Diels–Alder reaction (DA) and the ring-rearrangement metathesis (RRM) have been used to design new polycycles. In this regard, ruthenium alkylidene catalysts are effective in realizing the RRM of bis-norbornene derivatives prepared by DA reaction and Grignard addition. Here, fused polycycles are assembled which are difficult to produce by conventional synthetic routes.

## Introduction

**Figure 1:**
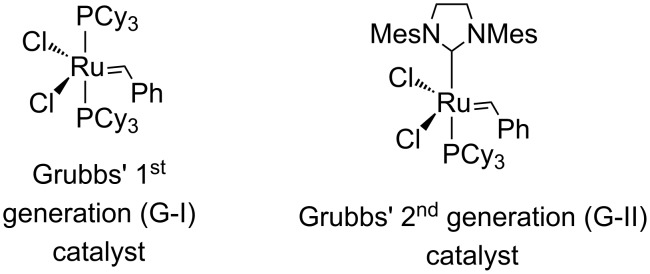
Commercially available ruthenium catalysts used in RRM metathesis.

Design and synthesis of complex polycycles in a minimum number of steps will enhance the overall synthetic economy of the preparation of a target molecule. The ring-rearrangement metathesis (RRM) is a conceptually novel, synthetically useful atom-economic method for the construction of complex molecules and by this process compounds containing several stereocenters are produced starting from simple starting materials. RRM involves a combination of two or more metathetic transformations, wherein multiple bond forming and bond breaking events take place in a one-pot operation [1–20]. Interestingly, the stereochemical information from the starting material is transferrred to the product. Moreover, RRM enables unprecedented and indirect routes to polycycles. For successful application of this strategy it is desirable that the starting materials have ring strain so that they can readily undergo a C=C double bond cleavage. Release of ring strain is the main driving force for RRM. In this regard, bicyclo[2.2.1] and bicyclo[2.2.2] systems are well suited. Here, we demonstrate that an endocyclic bis-norbornene system undergoes an RRM with a suitably placed olefin moiety to generate complex polycyclic compounds. RRM of norbornene derivatives are common, however, reports dealing with RRM of bis-norbornene derivatives are rare [21–22]. Herein, we report two unique examples where the synthesis of hexacyclic systems containing 10 stereocenters have been generated by the application of RRM of readily available bis-norbornene derivatives using Grubbs’ catalysts (Figure 1). The higher analogue related to the bicyclo[2.2.2] system is also studied.

## Results and Discussion

**Figure 2:**
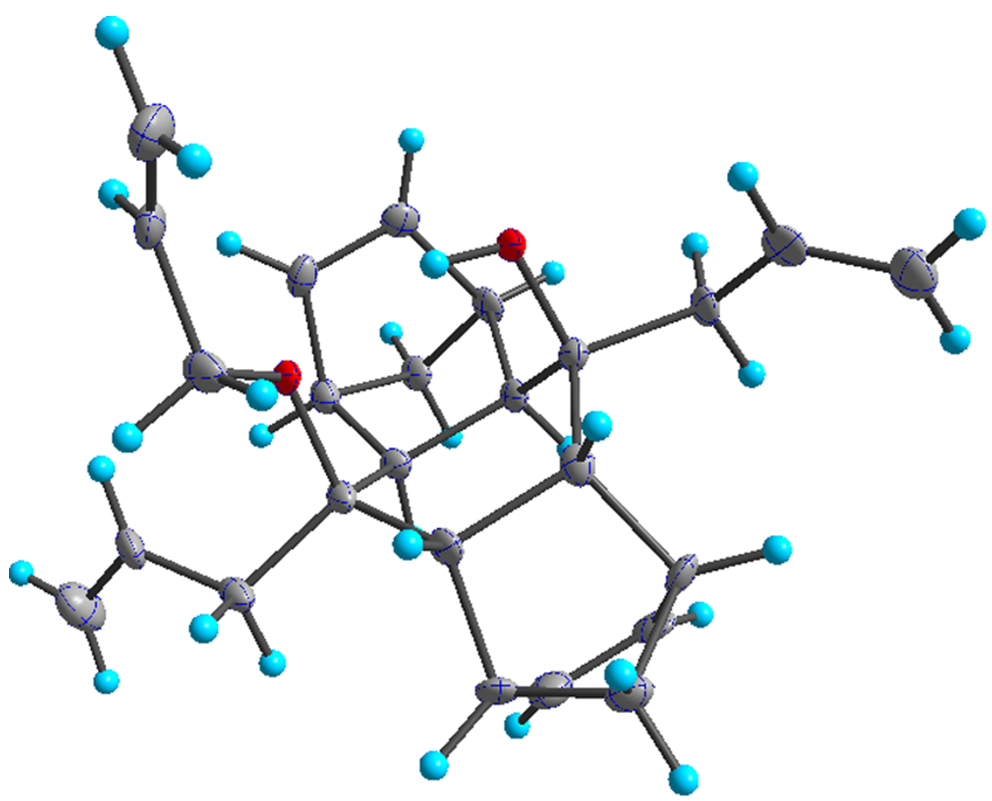
Crystal structure of **5** with thermal ellipsoids drawn at 50% probability level.

Our strategy to polycycles involves a Diels–Alder reaction (DA) [23–25], a Grignard addition [26] and a RRM as key steps. To begin with, a double DA reaction of cyclopentadiene (**1**) with 1,4-benzoquinone (**2**) gave the known bis-adduct **3** [27–28]. Later, it was reacted with allylmagnesium bromide to produce 1,2-addition product **4**. A molecular model of compound **3** reveals that its exo-face is more accessible for Grignard addition than the endo-face. Also, the X-ray structure of compound **5** indicates the stereostructure of **4**. Further, the diol **4** was treated with four equivalents of allyl bromide in the presence of an excess amount of NaH to generate the mono-*O*-allyl compound **5** and surprisingly the di-*O*-allyl compound was not formed. The stereostructure of **5** has been established on the basis of single-crystal X-ray diffraction studies [29] and it shows the steric hindrance associated with one of the hydroxy groups (Figure 2).

**Scheme 1:**
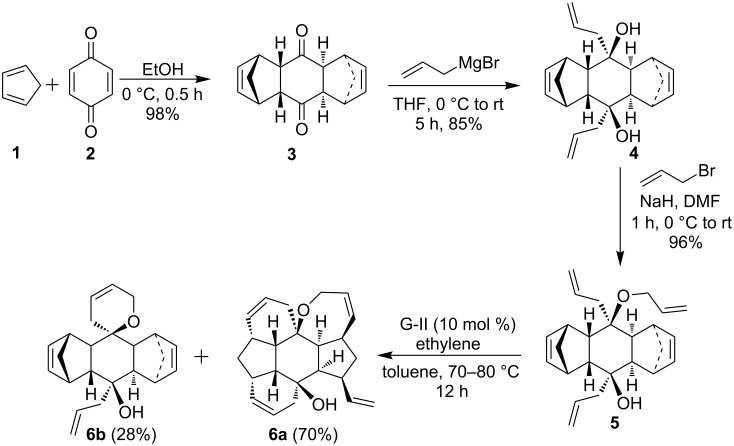
Synthesis of hexacyclic compound **6a** by using an RRM approach.

Later, the triallyl compound **5** was subjected to RRM in the presence of G-II catalyst (Figure 1) under ethylene atmosphere to deliver the hexacyclic rearranged product **6a** in 70% yield and ring-closing spiro product **6b** in 28% yield (Scheme 1).

**Scheme 2:**
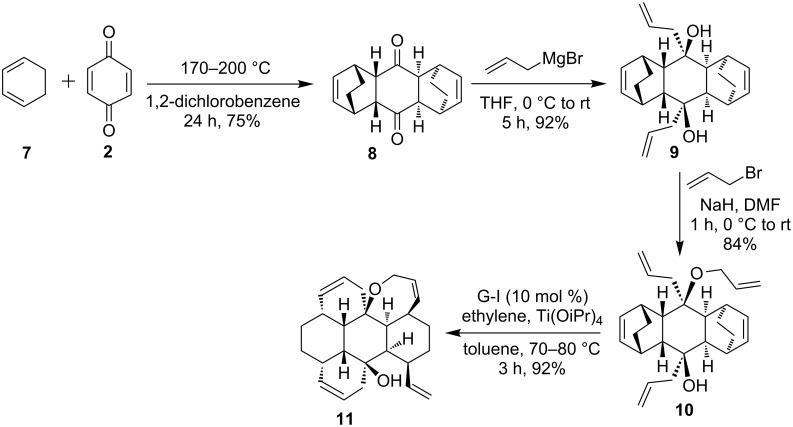
Synthesis of hexacyclic compound **11** by using an RRM route.

To expand this strategy, next we focussed on the preparation of an analogous bicyclo[2.2.2] system and to this end, the DA reaction of 1,3-cyclohexadiene (**7**) with 1,4-benzoquinone (**2**) furnished the known bis-adduct **8** [27–28], which on treatment with allylmagnesium bromide delivered diol **9**. Later, *O*-allylation of diol **9** with four equivalents of allyl bromide in the presence of NaH in DMF gave the mono *O*-allyl compound **10**. Attempts to achieve complete allylation of **10** were not successful. Finally, the RRM of compound **10** in the presence of G-I catalyst (Figure 1) under ethylene atmosphere gave the hexacyclic derivative **11** in 92% yield (Scheme 2). The structures of various polycyclic derivatives have been established on the basis of ^1^H and ^13^C NMR spectral data and further supported by HRMS data.

## Conclusion

We have demonstrated a simple, useful and atom-economic methodology for the synthesis of polycycles via DA reaction and RRM as key steps. Here, we generated polycyclic compounds with 10 stereocenters involving six fused rings in four steps starting with readily available starting materials such as 1,3-cyclopentadiene, 1,3-cyclohexadiene and 1,4-benzoquinone. Further studies to expand the scope of this strategy are underway. The strategy demonstrated here is likely to find useful applications in complex targets.

## Experimental

### General remarks

All reactions were monitored by employing thin layer chromatography (TLC) technique using an appropriate solvent system for development. Reactions involving oxygen-sensitive reagents or catalysts were performed in degassed solvents. Dry tetrahydrofuran (THF) and dry ether were obtained by distillation over sodium benzophenone ketyl freshly prior to use. Dichloromethane (DCM) and toluene were distilled over P_2_O_5_ and DMF over CaH_2_. Sodium sulfate was dried in an oven at 130 °C for one day. All solvent extracts were washed successively with water and brine (saturated sodium chloride solution), dried over anhydrous sodium sulfate, and concentrated at reduced pressure on a rotary evaporator. Yields refer to the chromatographically isolated sample. All the commercial grade reagents were used without further purification. NMR samples were generally made in chloroform-*d* solvent, and chemical shifts were reported in δ scale using tetramethylsilane (TMS) as an internal standard. The standard abbreviations s, d, t, q and m, refer to singlet, doublet, triplet, quartet, and multiplet, respectively. Coupling constants (*J*) are reported in Hertz.

### Experimental procedures

#### Synthesis of compound **4**

Analogously as described in [2], to a stirred solution of diketone **3** (0.2 g, 0.83 mmol) in dry THF (10 mL) was added allylmagnesium bromide (4.2 mL, 1 M solution in ether) at 0 °C under nitrogen atmosphere, and the reaction mixture was stirred for 5 h at rt. After completion of the reaction (TLC monitoring), the reaction mixture was quenched with saturated ammonium chloride and extracted with ethyl acetate. The combined organic layer was washed with water, brine and dried over sodium sulfate. The organic layer was concentrated under reduced pressure and the crude product was purified by silica gel column chromatography by eluting with 5% ethyl acetate in petroleum ether to afford **4** as a white solid (0.23 g, 85%). mp 130–131 °C; ^1^H NMR (500 MHz, DMSO) δ 6.12 (s, 2H), 6.05–5.98 (m, 2H), 5.92 (s, 2H), 5.14 (d, *J* = 17.1 Hz, 2H), 5.06 (d, *J* = 10.2 Hz, 2H), 4.68 (s, 2H), 2.78 (d, *J* = 16.8 Hz, 4H), 2.48–2.42 (m, 2H), 2.26 (s, 2H), 2.15 (dd, *J* = 14.3, 8.3 Hz, 2H), 1.61 (s, 2H), 1.19 (d, *J* = 8.0 Hz, 1H), 1.11 (d, *J* = 7.6 Hz, 2H), 0.99 (d, *J* = 7.3 Hz, 1H) ppm; ^13^C NMR (125 MHz, CDCl_3_) δ 135.5, 134.7, 134.4, 118.3, 73.4, 52.3, 52.3, 50.6, 49.2, 45.8, 45.7, 44.8 ppm; HRMS (Q–ToF) *m*/*z*: [M + Na]^+^ calcd for C_17_H_20_ONa, 347.1982; found, 347.1980.

#### Synthesis of compound **9**

Analogously as described in [2], to a stirred solution of diketone **8** (0.5 g, 1.8 mmol) in dry THF (10 mL) was added allylmagnesium bromide (11 mL, 1 M solution in ether) at 0 °C under nitrogen atmosphere, and the reaction mixture was stirred for 5 h at rt. After completion of the reaction (TLC monitoring), the reaction mixture was quenched with saturated ammonium chloride and extracted with ethyl acetate. The combined organic layer was washed with water, brine and dried over sodium sulfate. The organic layer was concentrated under reduced pressure and the crude product was purified by silica gel column chromatography by eluting with 10% ethyl acetate in petroleum ether to afford **9** as a white solid (0.6 g, 92%). mp 122–125 °C; ^1^H NMR (400 MHz, CDCl_3_) δ 6.29 (dd, *J* = 4.7, 3.3 Hz, 2H), 6.18 (dd, *J* = 4.6, 3.3 Hz, 2H), 6.05–5.95 (m, 2H), 5.16 (dd, *J* = 5.7, 1.4 Hz, 4H), 3.78 (s, 2H), 2.71 (d, *J* = 14.2 Hz, 4H), 2.62 (dd, *J* = 14.6, 6.2, 2H), 2.26 (dd, *J* = 14.6, 7.5, 2H), 2.07 (s, 2H), 1.63 (s, 2H), 1.42 (t, *J* = 6.9 Hz, 4H), 1.24–1.21 (m, 2H), 1.15–1.12 (m, 2H) ppm; ^13^C NMR (100 MHz, CDCl_3_) δ 134.9, 134.2, 132.4, 117.9, 73.7, 49.2, 48.7, 44.7, 32.4, 31.1, 26.6, 26.4 ppm; HRMS (Q–ToF) *m*/*z*: [M + Na]^+^ calcd for C_24_H_32_O_2_Na, 375.2295; found, 375.2293.

#### Synthesis of compound **5**

Analogously as described in [2], to a suspension of NaH (26 mg, 1.08 mmol) in dry DMF (10 mL), was added solution of compound **4** (50 mg, 0.15 mmol) in DMF (5 mL) and allyl bromide (0.074 g, 0.62 mmol) at 0 °C under nitrogen atmosphere and stirred at rt for 1 h. After completion of the reaction (TLC monitoring), the reaction mixture was quenched with saturated ammonium chloride and extracted with ethyl acetate. The combined organic layer washed with water, brine dried over sodium sulfate. The organic layer was concentrated under reduced pressure and purified by silica gel column chromatography by eluting with 5% ethyl acetate in petroleum ether to afford **5** as a white solid (60 mg, 96%). mp 105–108 °C; ^1^H NMR (500 MHz, CDCl_3_) δ 6.09–6.17 (m, 2H), 6.05–5.97 (m, 3H), 5.93 (dd, *J =* 5.4, 3.0 Hz, 1H), 5.84–5.76 (m, 1H), 5.22 (dq, *J =* 17.2, 1.5 Hz, 1H), 5.19–5.17 (m, 1H), 5.17–5.07 (m, 4H), 4.43 (d, *J =* 1.9 Hz, 1H), 3.94 (dd, *J =* 11.6, 5.7 Hz, 1H), 3.85–3.82 (m, 1H), 3.00 (s, 1H), 2.94 (s, 1H), 2.84 (s, 1H), 2.79 (s, 1H), 2.76–2.70 (m, 1H), 2.65 (dd, *J =* 9.8, 3.5 Hz, 1H), 2.54 (dd, *J =* 14.2, 6.2 Hz, 1H), 2.41 (dd, *J =* 9.8, 3.5 Hz, 1H), 2.32 (dd, *J =* 15.6, 8.2 Hz, 1H), 2.18–2.13 (m, 1H), 1.75–1.73 (m, 2H), 1.42–1.39 (m, 1H), 1.28 (d, *J =* 4.0 Hz, 1H), 1.22–1.20 (m, 1H), 1.0 (d, *J =* 7.7 Hz, 1H) ppm; ^13^C NMR (125 MHz, CDCl_3_) δ 135.9, 135.3, 135.1, 134.9, 134.4, 134.1, 133.5, 117.0, 116.9, 116.6, 79.4, 72.1, 62.6, 52.4, 51.6, 51.4, 49.6, 49.5, 45.9, 45.7, 45.6, 45.5, 45.1, 44.4, 42.5 ppm; HRMS (Q–ToF) *m*/*z*: [M + Na]^+^ calcd for C_25_H_32_O_2_Na, 387.2295; found, 387.2295.

#### Synthesis of compound **10**

Analogously as described in [2], to a suspension of NaH (115 mg, 4.77 mmol) in dry DMF (10 mL), was added a solution of compound **9** (240 mg, 0.68 mmol) in DMF (10 mL) and allyl bromide (0.33 g, 2.72 mmol) at 0 °C under nitrogen atmosphere and stirred at rt for 1 h. After completion of the reaction (TLC monitoring), the reaction mixture was quenched with saturated ammonium chloride and extracted with ethyl acetate. The combined organic layer was washed with water, brine and dried over sodium sulfate. The organic layer was concentrated under reduced pressure and purified by silica gel column chromatography by eluting with 5% ethyl acetate in petroleum ether to afford **11** as a yellow semisolid (224 mg, 84%). ^1^H NMR (400 MHz, CDCl_3_) δ 6.22–6.18 (m, 1H), 6.17–6.07 (m, 3H), 6.07–6.00 (m, 1H), 5.99–5.92 (m, 1H), 5.84–5.73 (m, 1H), 5.31 (d, *J =* 2.5 Hz, 1H), 5.27 (dq *J =* 17.2, 1.7 Hz, 1H), 5.20–5.14 (m, 1H), 5.13–5.06 (m, 3H), 3.96–3.87 (m, 2H), 2.80–2.56 (m, 7H), 2.32 (d, *J =* 1.5 Hz, 1H), 2.21–2.14 (m, 1H), 2.40 (d, *J =* 1.6 Hz, 1H), 1.67 (d, *J =* 7.0 Hz, 2H), 1.58 (d, *J =* 7.5 Hz, 1H), 1.57–1.42 (m, 2H), 1.31–1.41 (m, 2H), 1.26–1.11 (m, 4H) ppm; ^13^C NMR (100 MHz, CDCl_3_) δ 136.0, 135.5, 134.3, 132.9, 132.6, 132.4, 131.9, 117.1, 116.6, 116.4, 79.9, 71.9, 62.4, 50.4, 49.7, 48.6, 44.9, 44.4, 42.9, 32.3, 31.9, 30.7, 30.6, 27.5, 26.7, 26.1 ppm; HRMS (Q–ToF) *m*/*z*: [M + Na]^+^ calcd for C_27_H_36_O_2_Na, 415.2608; found, 415.2605.

#### Synthesis of compounds **6a** and **6b**

Analogously as described in [2], to a stirred solution of compound **5** (40 mg, 0.11 mmol) in toluene (40 mL) degassed with nitrogen for 10 minutes, purged with ethylene gas for another 10 minutes and then G-II catalyst (8 mg, 10 mol %) was added and stirred at 70 °C for 12 h under ethylene atmosphere. After completion of the reaction (TLC monitoring), the solvent was removed on a rotavapor under reduced pressure and purified by silica gel column chromatography by eluting with 5–10% ethyl acetate in petroleum ether provided **6a** and **6b** as a colourless liquids (25 mg and 10 mg, 70% and 28%, respectively). **6a**; ^1^H NMR (500 MHz, CDCl_3_) δ 6.31–6.24 (m, 1H), 6.10 (dd, *J* = 5.6, 3.0 Hz, 1H), 6.05 (dd, *J* = 5.6, 2.7 Hz, 1H), 5.85 (dt, *J* = 9.8, 2.8 Hz, 1H), 5.80–5.76 (m, 1H), 5.68–5.65 (m, 2H), 4.99 (dd, *J* = 17.1, 2.4 Hz, 1H), 4.88 (dd, *J* = 9.9, 2.4 Hz, 1H), 4.33–4.29 (m, 1H), 4.22–4.18 (m, 1H), 2.95–2.88 (m, 2H), 2.82 (s, 1H), 2.68–2.61 (m, 2H), 2.56 (dd, *J* = 10.1, 3.5 Hz, 1H), 2.35–2.29 (m, 1H), 2.21–1.92 (m, 3H), 1.85–1.60 (m, 5H), 1.44 (dt, *J* = 7.9, 1.8 Hz, 1H), 1.35 (d, *J* = 8.0 Hz, 1H) ppm; ^13^C NMR (125 MHz, CDCl_3_) *δ* = 142.5, 135.3, 132.5, 129.2, 123.9, 123.8, 123.1, 114.3, 76.2, 68.4, 60.2, 52.3, 50.7, 49.6, 47.6, 46.7, 45.9, 42.7, 41.2, 41.1, 40.9, 37.0, 32.3 ppm; HRMS (Q–ToF) *m*/*z*: [M + Na]^+^ calcd for C_25_H_32_NaO_2_, 359.1982; found, 359.1988; IR (neat) *ν*_max_: 3050, 2954, 1691, 1610, 1266 cm^−1^.

**6b**; ^1^H NMR (500 MHz, CD_3_OD) δ 6.23 (dd, *J* = 5.6, 3.0 Hz, 1H), 6.17 (dd, *J* = 5.7, 2.8 Hz, 1H), 6.15–6.08 (m, 1H), 5.95 (t, *J* = 2.4 Hz, 2H), 5.99–5.89 (m, 1H), 5.72 (dd, *J* = 10.3, 2.4 Hz, 1H), 5.22 (dd, *J* = 17.1, 1.4 Hz, 1H), 5.14 (d, *J* = 10.2 Hz, 1H), 4.22–4.10 (m, 2H), 2.97 (s, 1H), 2.93 (dd, *J* = 10.0, 3.7 Hz, 1H), 2.87 (s, 1H), 2.68 (s, 1H), 2.59–2.53 (m, 2H), 2.48 (dd, *J* = 10.0, 3.4 Hz, 1H), 2.29 (dd, *J* = 14.4, 8.0 Hz, 1H), 2.09–2.03 (m, 1H), 1.90–1.84 (m, 2H), 1.43–1.41 (m, 1H), 1.35–1.30 (m, 4H), 1.15 (d, *J* = 7.6 Hz, 1H) ppm; ^13^C NMR (125 MHz, CDCl_3_) δ 134.8, 134.7, 133. 9, 133.5, 124.1, 122.6, 116.0, 73.8, 73.1, 59.9, 51.5, 51.1, 50.9, 49.5, 45.6, 44.6, 44.5, 44.1, 40.1, 32.1 ppm; HRMS (Q–ToF) *m*/*z*: [M + Na]^+^ calcd for C_25_H_32_NaO_2_, 359.1982; found, 359.1986.

#### Synthesis of compound **11**

Analogously as described in [2], to a stirred solution of compound **10** (20 mg, 0.05 mmol) in toluene (20 mL) degassed with nitrogen for 10 minutes, purged with ethylene gas for another 10 minutes and then added titanium isopropoxide and G-I catalyst (4 mg, 10 mol %) and stirred at 70–80 °C for 3 h under ethylene atmosphere. After completion of the reaction (TLC monitoring), solvent was removed on rotavapor under reduced pressure and purified by silica gel column chromatography by eluting with 5% ethyl acetate in petroleum ether provided **11** as a yellow semisolid (17 mg, 92%). ^1^H NMR (500 MHz, CDCl_3_) δ 6.24 (t, *J* = 7.1 Hz, 1H), 6.24–6.11 (m, 2H), 6.07–5.99 (m, 2H), 5.89–5.84 (m, 1H), 5.63 (dq , *J* = 10.2, 2.6 Hz, 1H), 5.15–5.08 (m, 3H), 4.19–4.07 (m, 2H), 2.71–2.57 (m, 5H), 2.42–2.34 (m, 2H), 2.24–2.18 (m, 1H), 2.05 (d, *J* = 10.1 Hz, 1H), 1.99–1.94 (m, 1H), 1.62–1.54 (m, 2H), 1.52–1.45 (m, 1H), 1.44–1.36 (m, 3H), 1.30–1.17 (m, 4H) ppm; ^13^C NMR (125 MHz, CDCl_3_) δ 135.8, 133.8, 133.1, 132.1, 131.2, 124.9, 123.4, 116.7, 74.7, 72.5, 61.1, 49.4, 49.2, 49.1, 44.5, 39.6, 34.2, 32.3, 31.5, 31.2, 30.6, 28.1, 27.4, 26.1, 25.3 ppm; HRMS (Q–ToF) *m*/*z*: [M + Na]^+^ calcd for C_25_H_32_NaO_2_, 387.2295; found, 387.2292.

## Supporting Information

File 1 Copies of ^1^H and ^13^C NMR spectra of new compounds; X-ray crystallographic data for compound **5**.
