# Supplementary material for: Design and synthesis of fused polycycles via Diels–Alder reaction and ring-rearrangement metathesis as key steps
Source: Beilstein J Org Chem. 2015 Jul 27;11:1259–64. doi: 10.3762/bjoc.11.140 (PMC4578369; doi:10.3762/bjoc.11.140)
Supplement: File 1 — Copies of 1H and 13C NMR spectra of new compounds; X-ray crystallographic data for compound 5. [file Beilstein_J_Org_Chem-11-1259-s001.pdf]

Supporting information

for

Design and synthesis of fused polycycles via Diels–Alder reaction and ring-rearrangement metathesis as key steps

Sambasivarao Kotha* and Ongolu Ravikumar

Address: Department of Chemistry, Indian Institute of Technology-Bombay, Powai, India,

Fax: 022-25767152

Email: Sambasivarao Kotha - srk@chem.iitb.ac.in

*Corresponding author

Copies of ^{1}H and ^{13}C NMR spectra of new compounds; X-ray crystallographic data for compound 5

Compound 4 ^{1}H and ^{13}C NMR (500 MHz, DMSO)

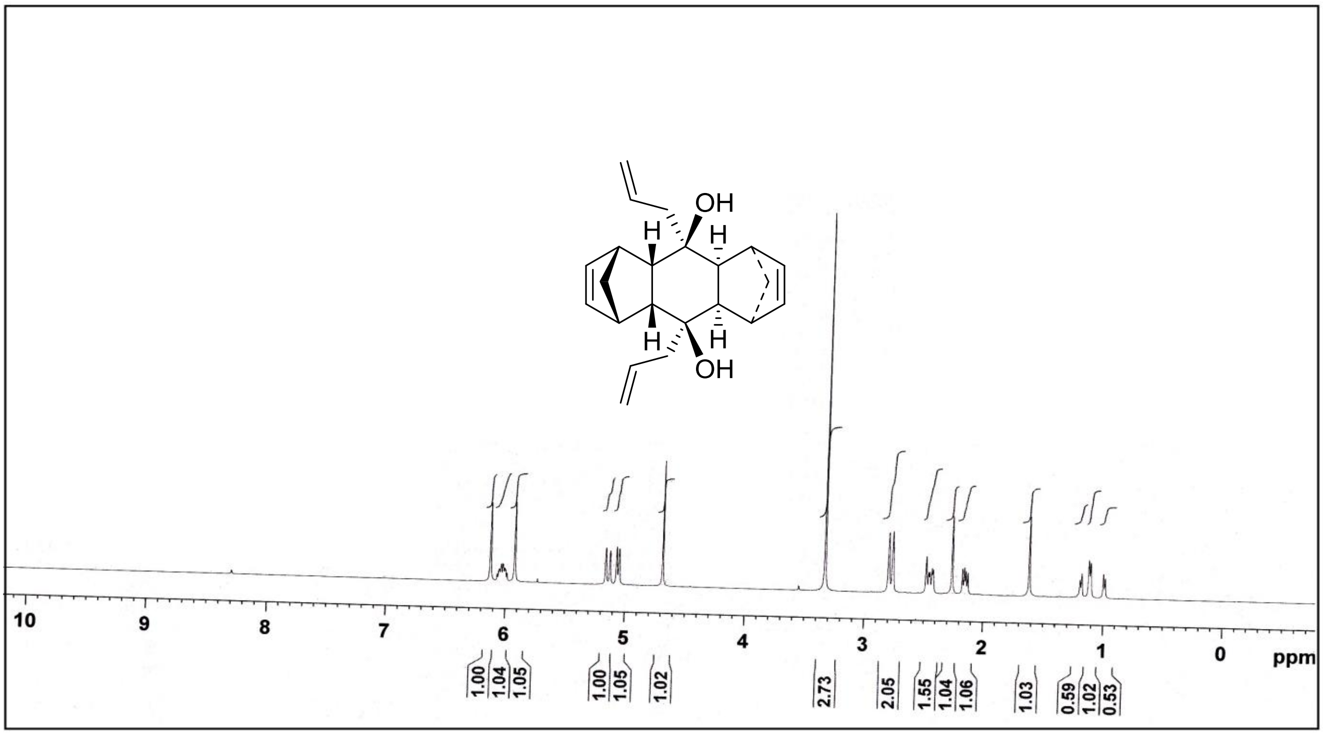

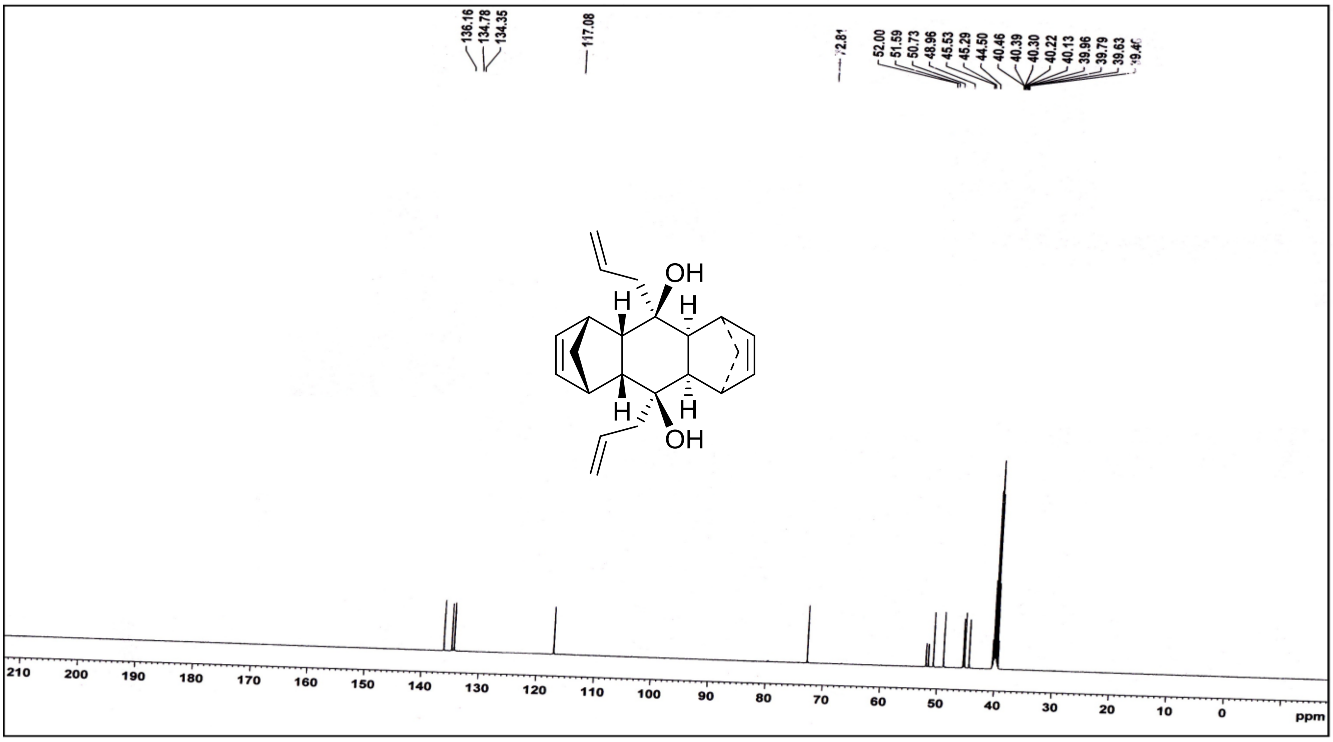

Compound 9 ^{1}H and ^{13}C NMR (400 MHz, $CDCl_3$)

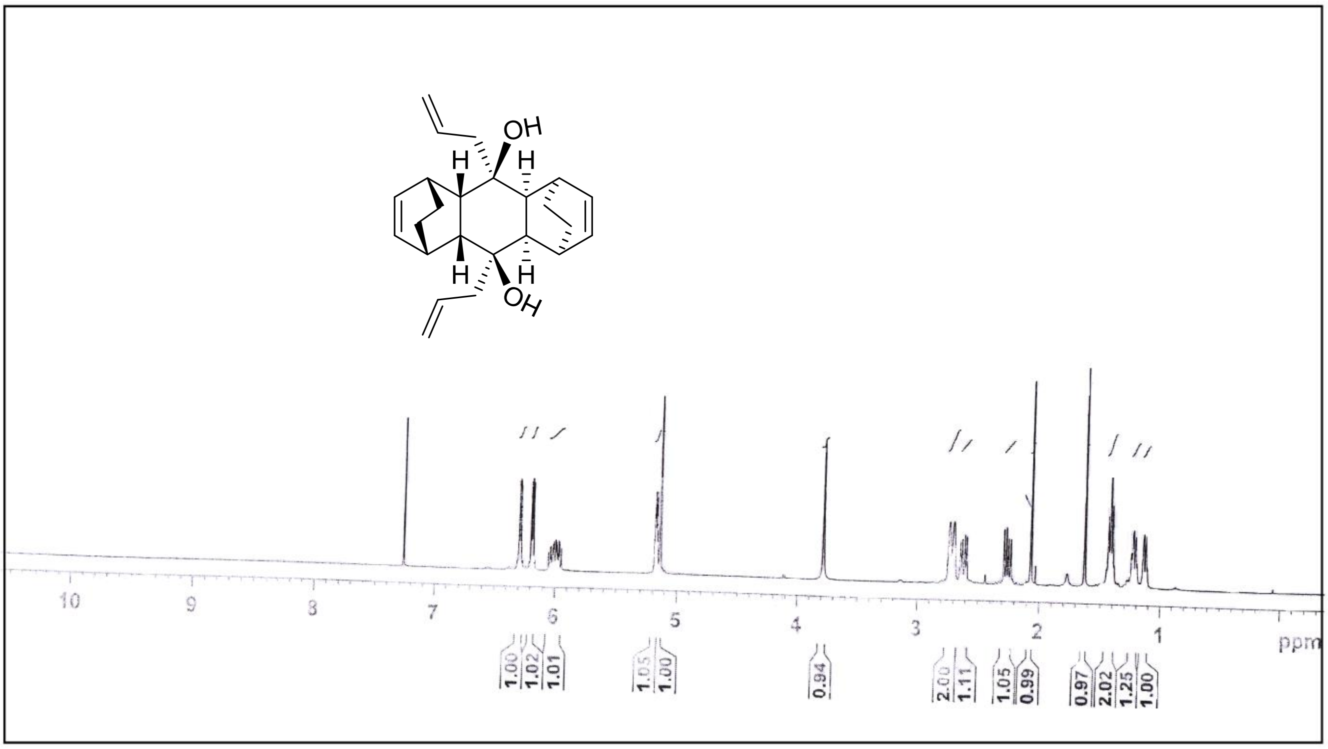

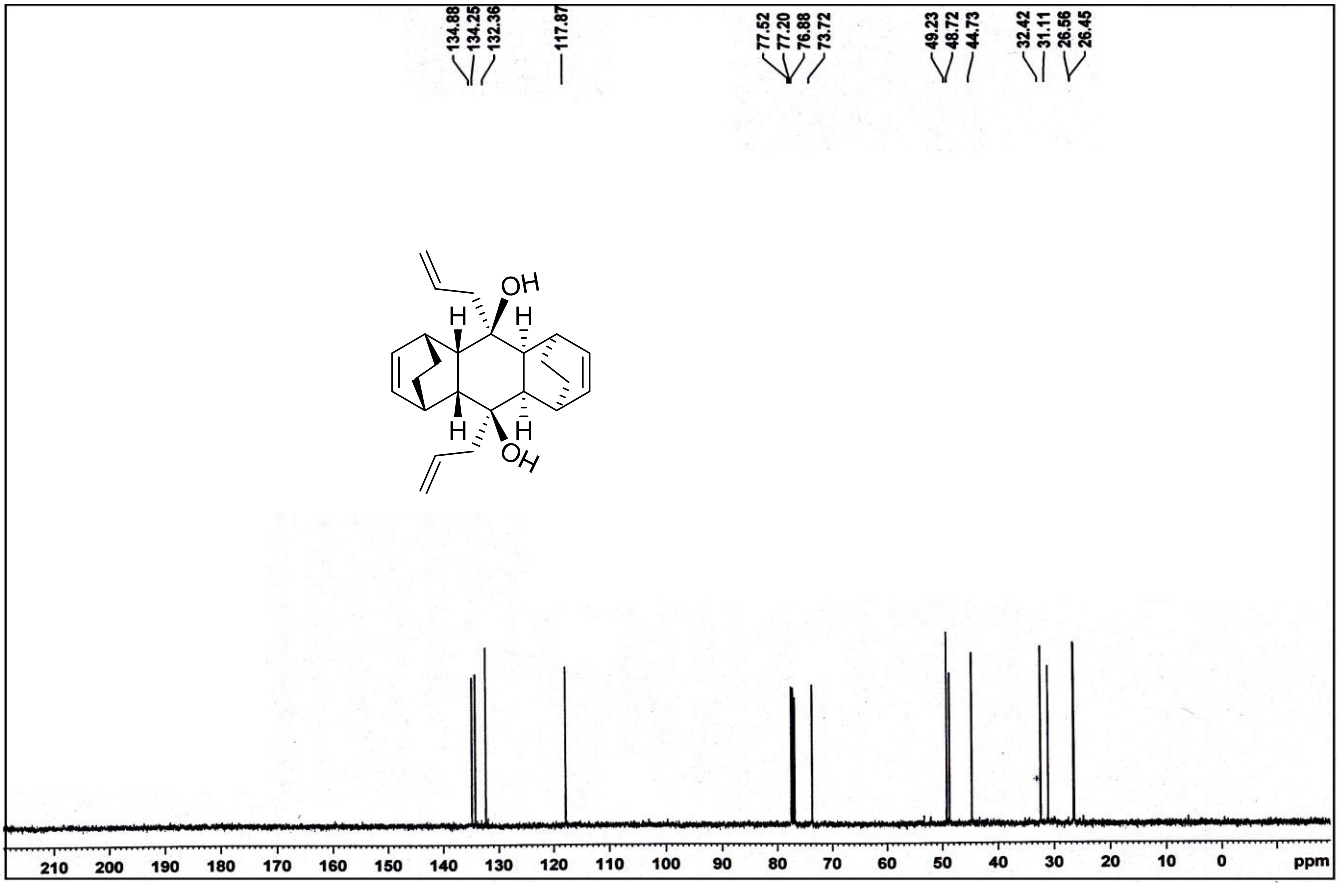

Compound 5 ^{1}H and ^{13}C NMR (500 MHz, $CDCl_3$)

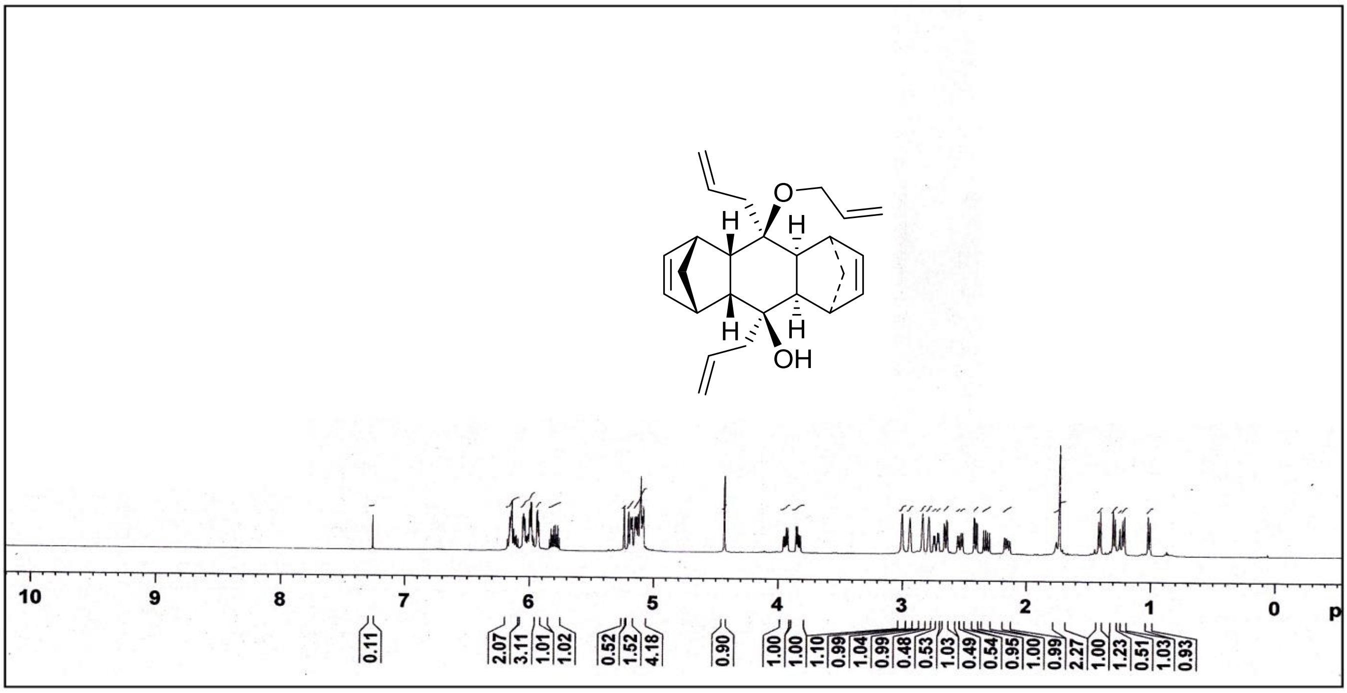

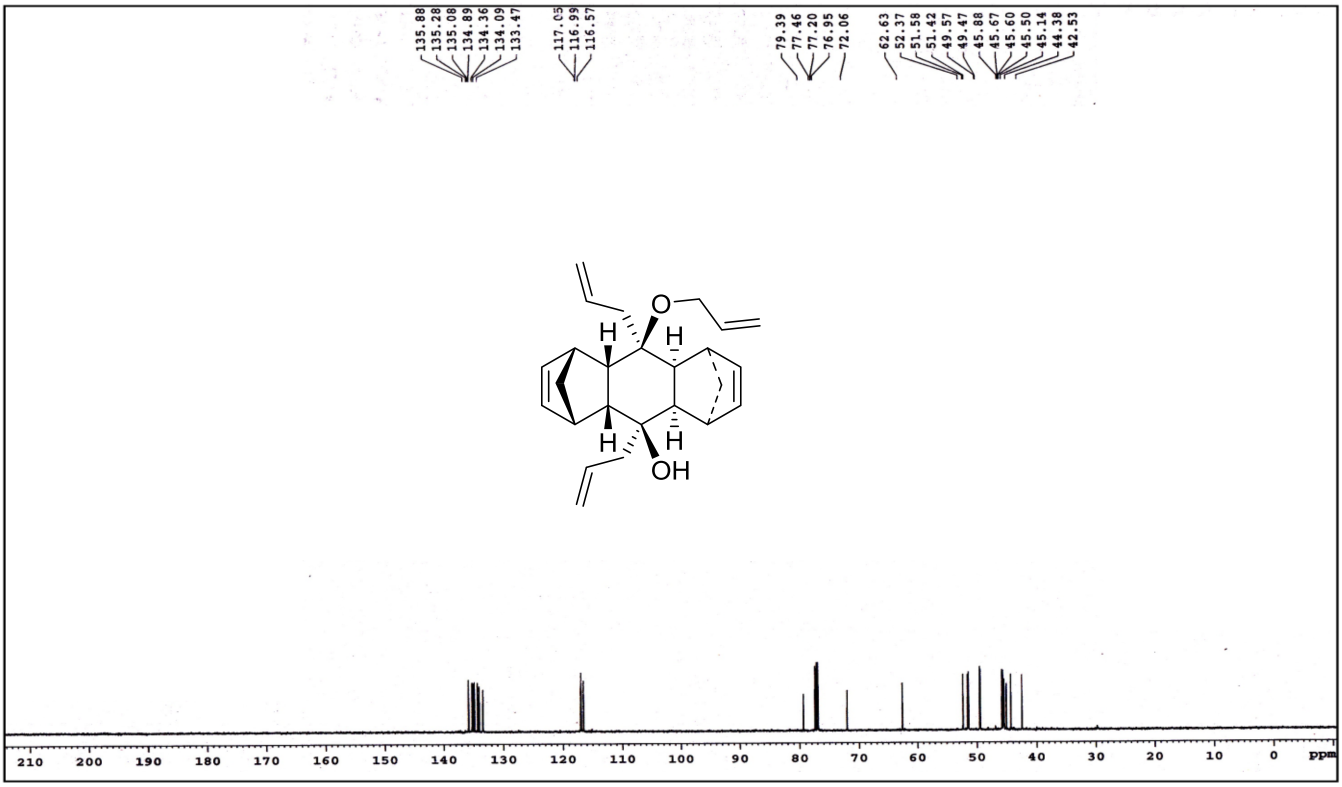

Compound 10 ^{1}H and ^{13}C NMR (400 MHz, $CDCl_3$)

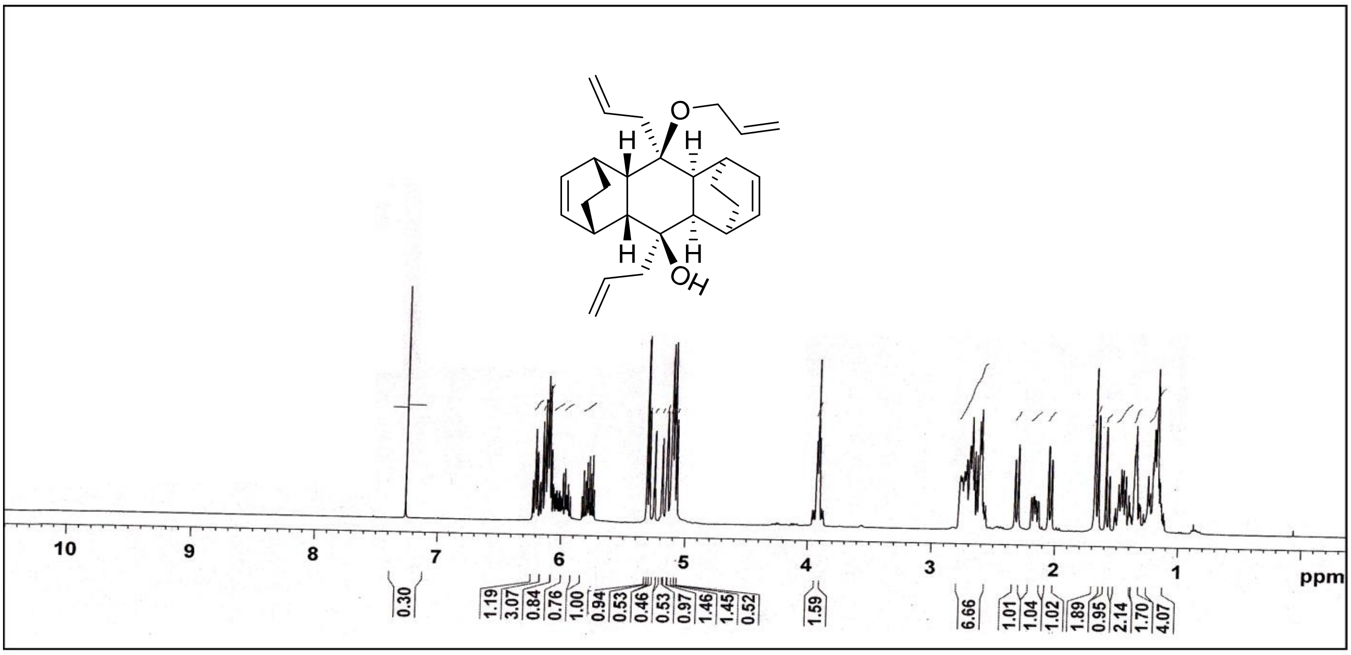

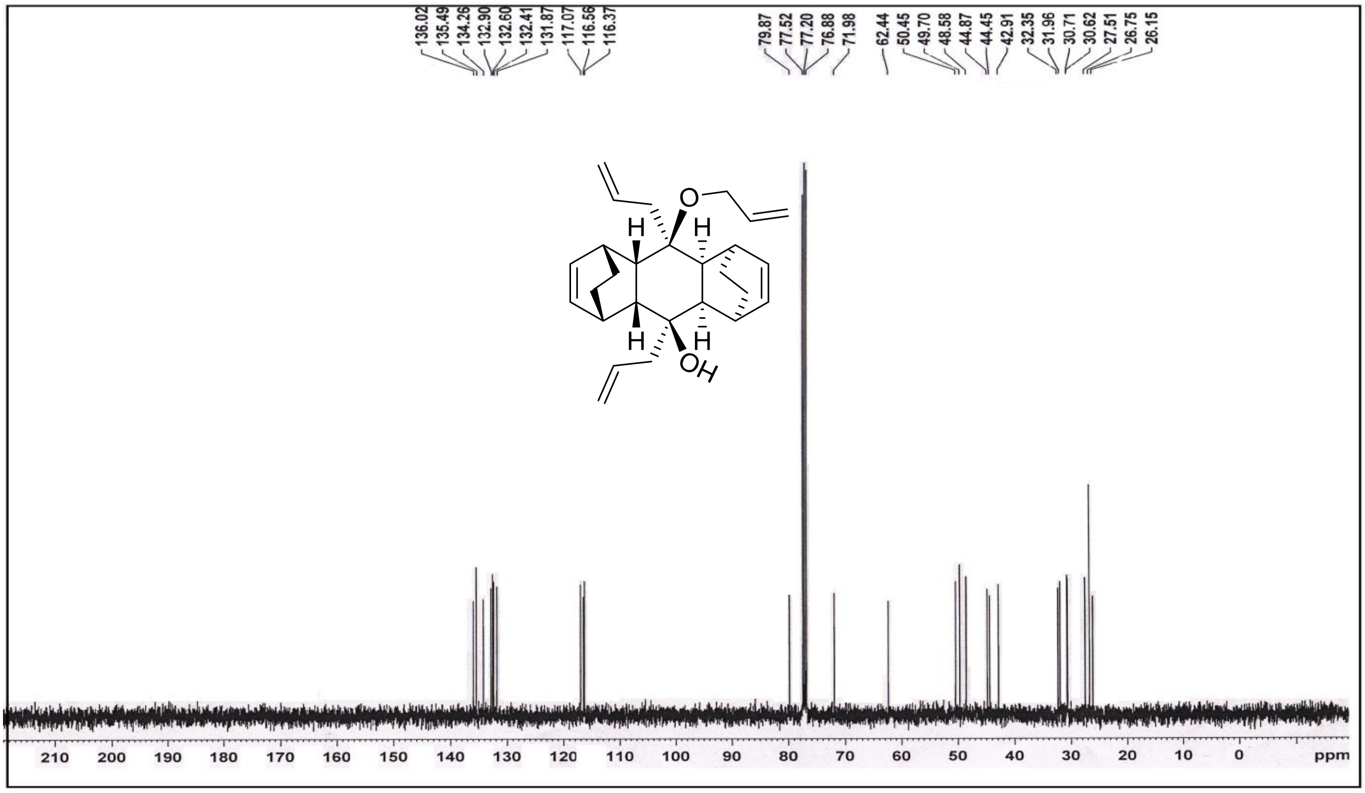

Compound 6a ^{1}H and ^{13}C NMR (500 MHz, $CDCl_3$)

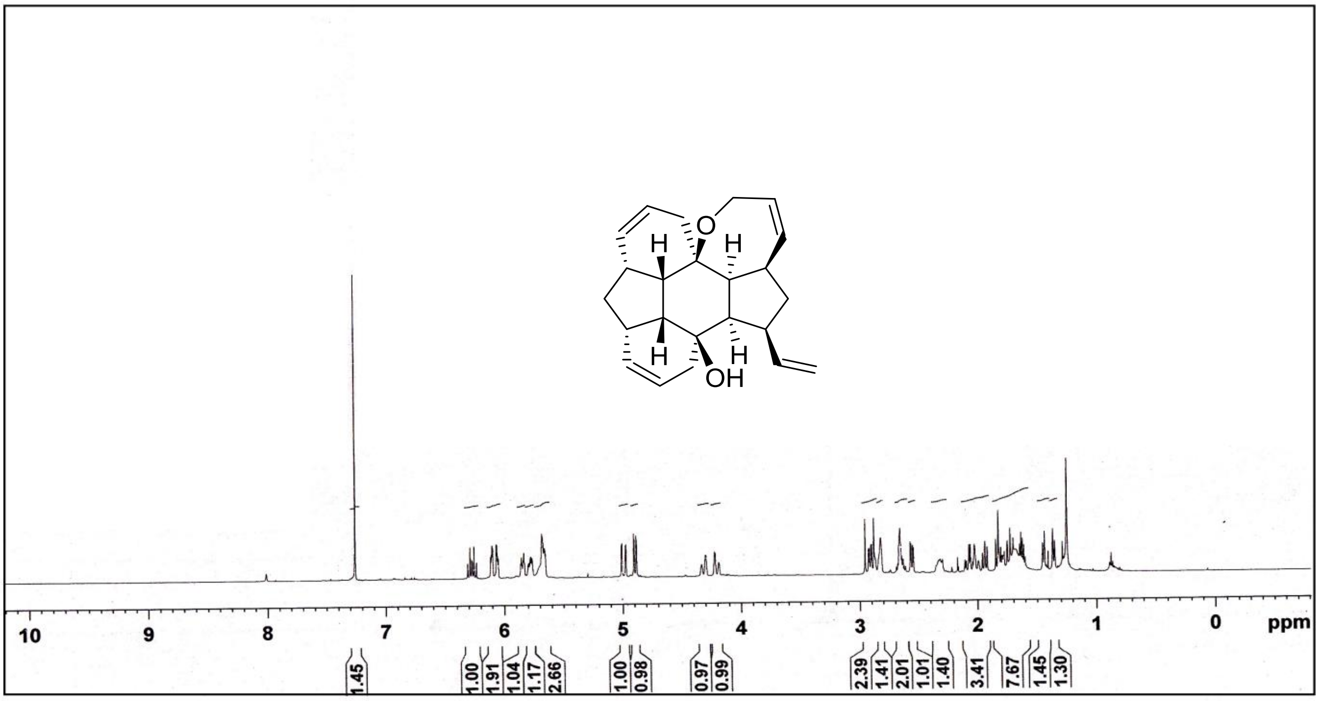

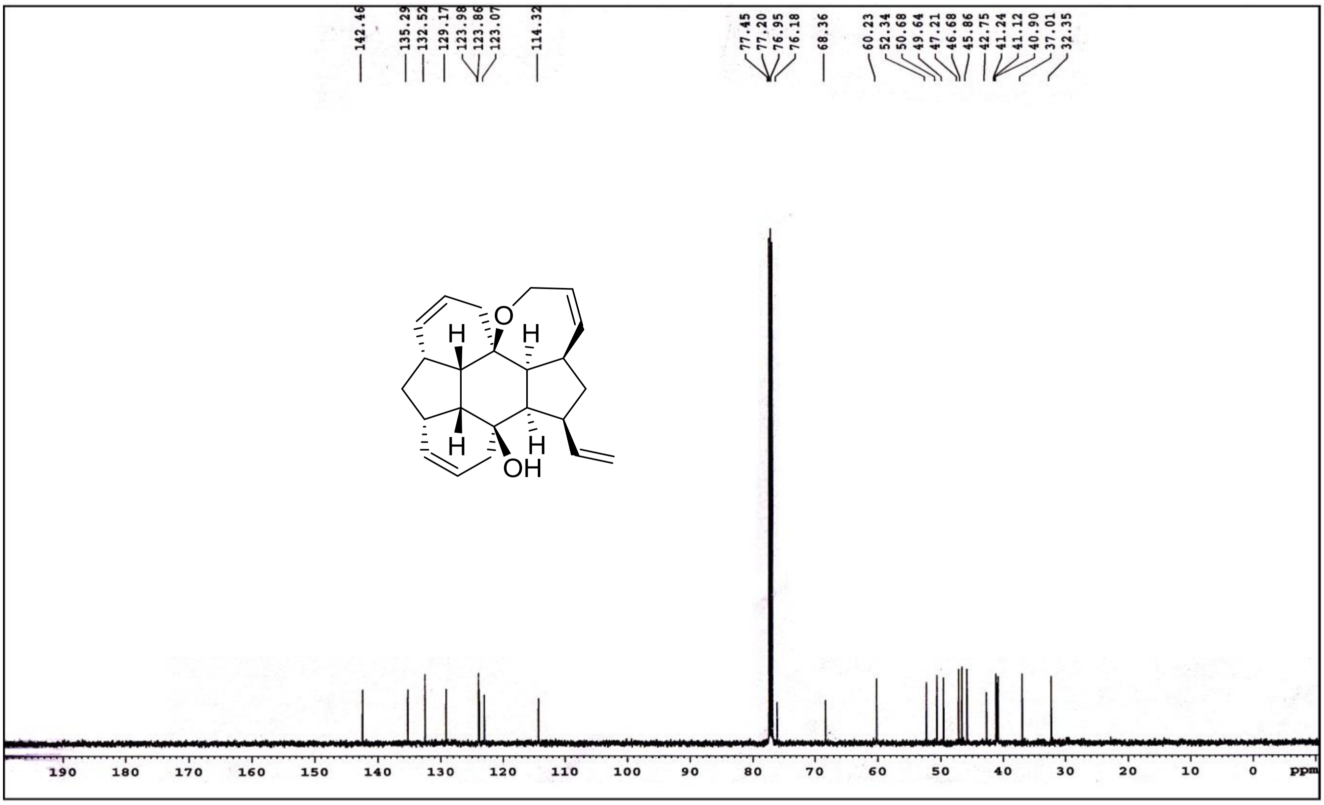

Compound 6b ^{1}H and ^{13}C NMR (500 MHz, MeOD)

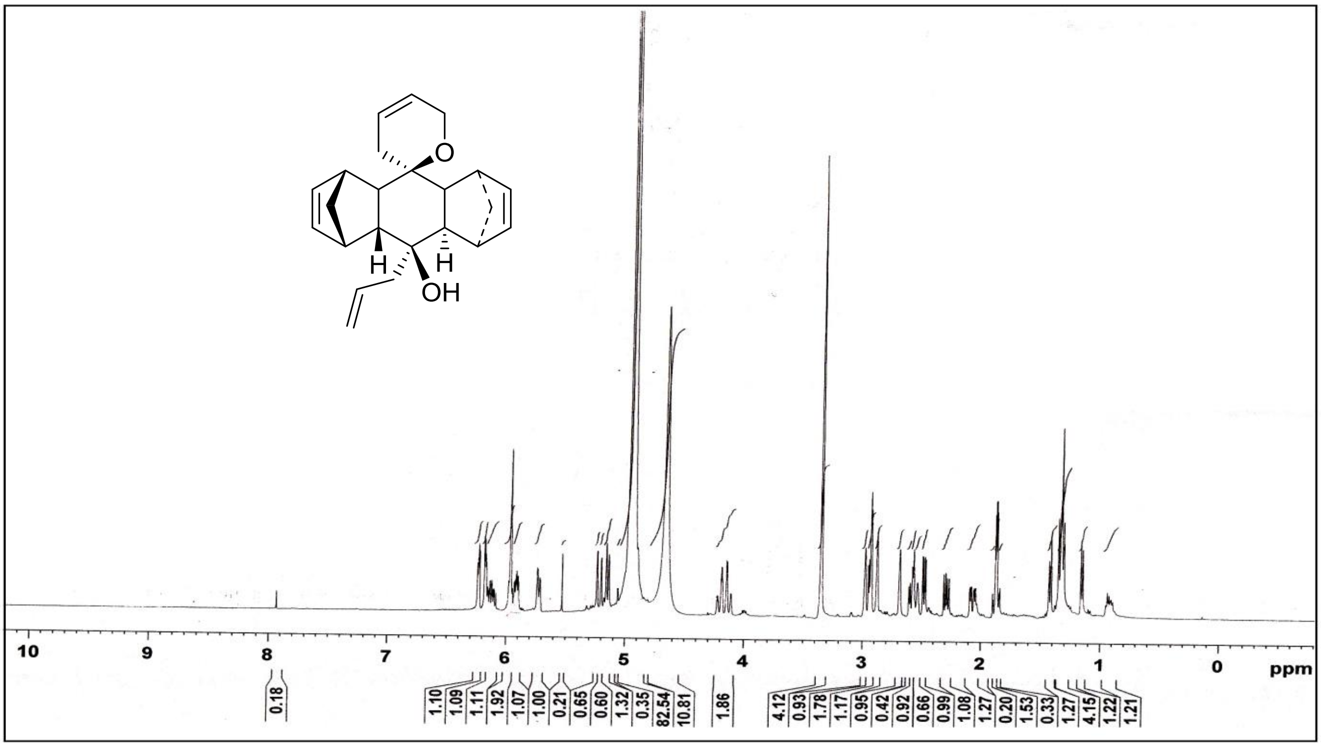

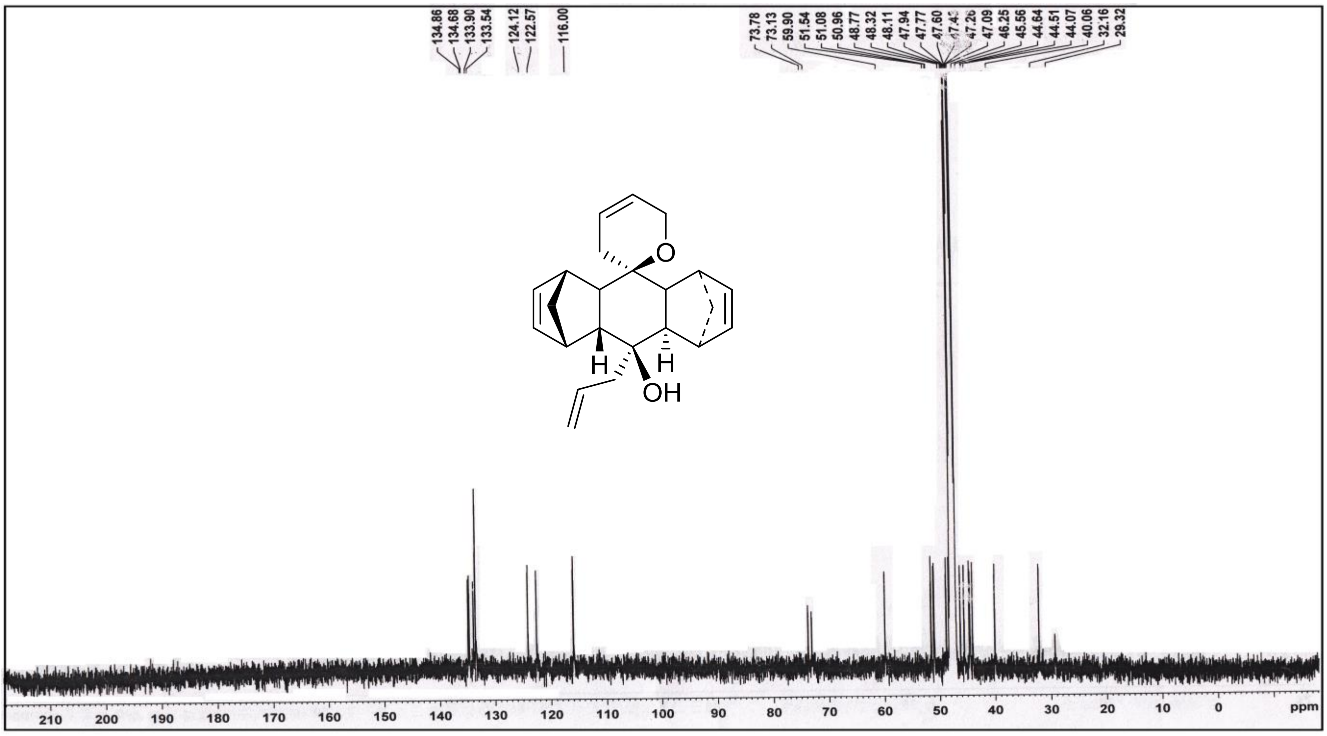

Compound 11 ^{1}H and ^{13}C NMR (500 MHz, $CDCl_3$)

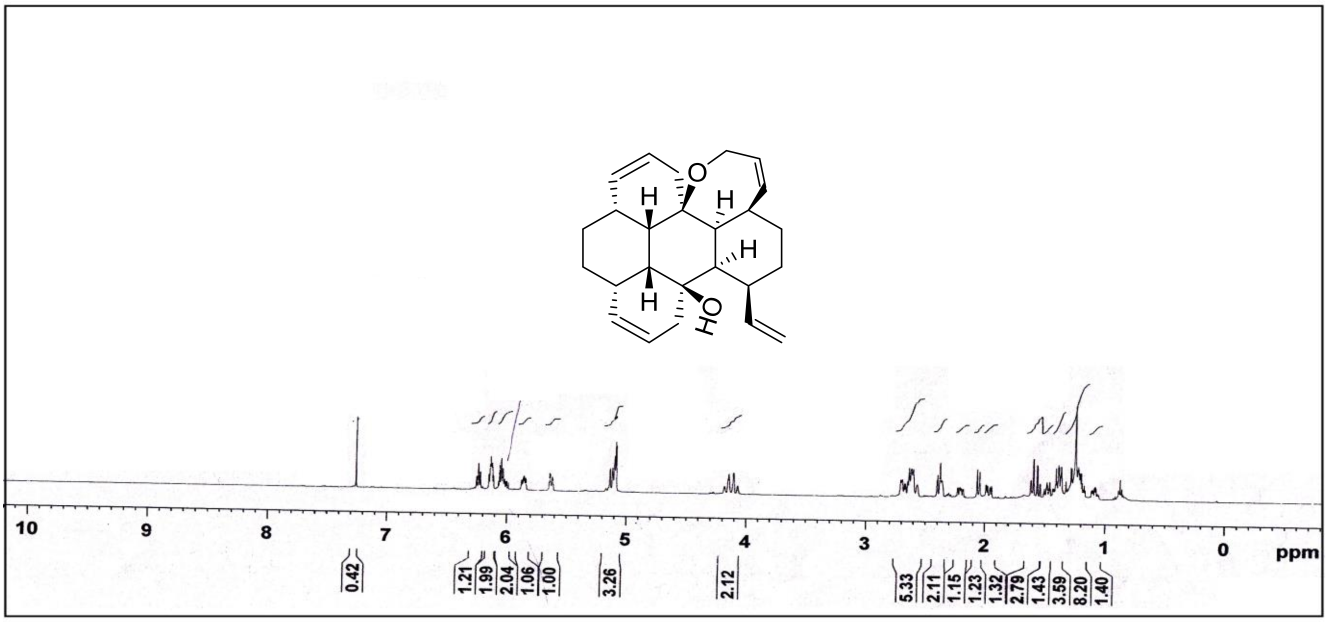

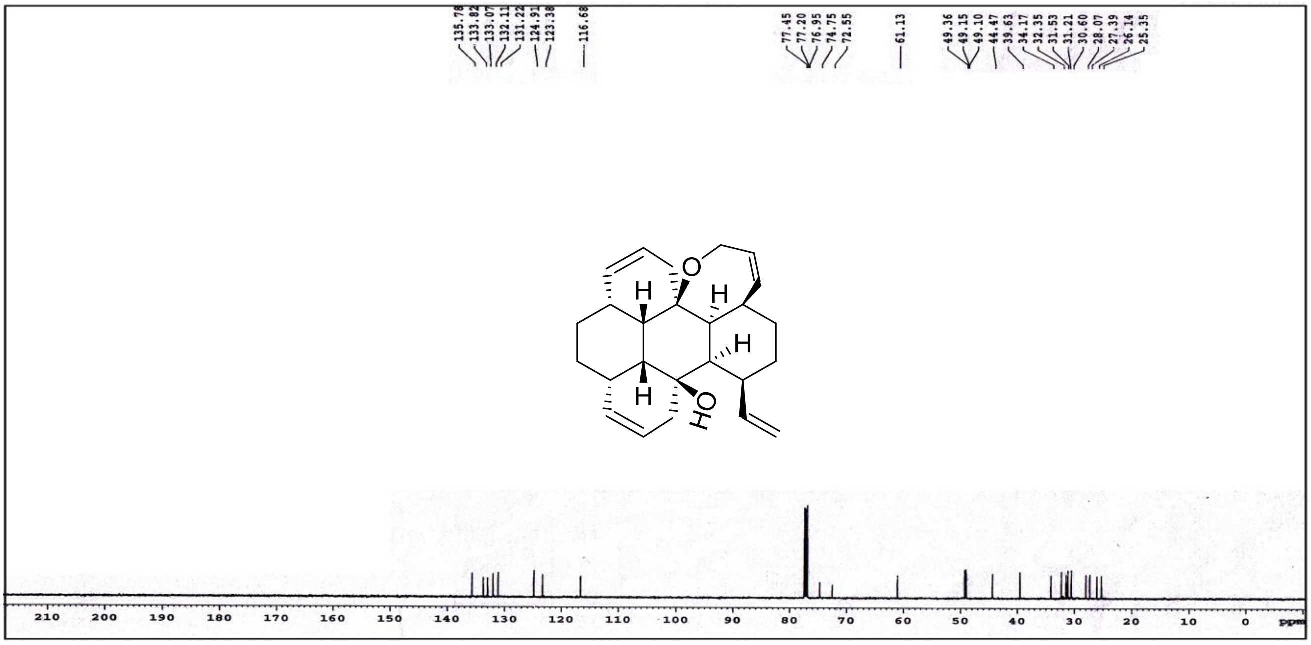

X-ray crystallographic data and refinement parameters for compound 5 (CCDC 1051925)

Bond precision: C-C = 0.0073 A Wavelength=0.71070

Cell:	a = 9.367(4)	b = 9.572(4)	c = 12.465(5)
	α = 71.456(18)	β = 79.53(2)	ɤ = 70.424(15)
Temperature:	100 K		

	**Calculated**	**Reported**
Volume	994.8 (7)	994.8 (8)
Space group	P -1	P -1
Hall group	-P 1	-P 1
Moiety formula	$C_{25} H_{32} O_2$	$C_{25} H_{32} O_2$
Sum formula	$C_{25} H_{32} O_2$	$C_{25} H_{32} O_2$
Mr	364.51	364.53
Dx,g cm^{-3}	1.217	1. 217
Z	2	2
Mu (mm-1)	0.075	0.075
F000	396.0	396.0
F000’	396.16	
h, k, lmax	11, 11, 14	11, 11, 14
Nref	3504	3486
Tmin, Tmax	0.982, 0.995	
Tmin’	0.981	

Correction method = Not given

Data completeness = 0.995 Theta (max) = 25.000

R(reflections) = 0.0782(1319) wR2(reflections) = 0.2821(3486)

S = 0.849 Npar = 244
